# Advancing the scientific study of prehospital mass casualty response through a Translational Science process: the T1 scoping literature review stage

**DOI:** 10.1007/s00068-023-02266-0

**Published:** 2023-04-15

**Authors:** Eric S. Weinstein, Joseph L. Cuthbertson, Teri Lynn Herbert, George T. Voicescu, Michelangelo Bortolin, Sabina Magalini, Daniele Gui, Mariana Helou, Kristina Lennquist Montan, Carl Montan, Chaim Rafalowsky, Giuseppe Ratto, Stefano Damele, Simone Bazurro, Itamar Laist, Federica Marzi, Alessandro Borrello, Pietro Fransvea, Andrea Fidanzio, Carlos Yanez Benitez, Roberto Faccincani, Luca Ragazzoni, Marta Caviglia

**Affiliations:** 1 CRIMEDIM—Center for Research and Training in Disaster Medicine, Humanitarian Aid, and Global Health, Università del Piemonte Orientale, Novara, Italy; 2 Research and Education Services, Medical University of South Carolina Library, Charleston, SC USA; 3 Department of Surgery, Policlinico Gemelli, Catholic University of the Sacred Heart, Rome, Italy; 4 School of Medicine, Department of Emergency Medicine, Lebanese American University, Beirut, Lebanon; 5 MRMID—International Association for Medical Response to Major Incidents and Disasters, and Vascular Surgery, Department of Molecular Medicine and Surgery, Karolinska Institutet, Stockholm, Sweden; 6 Magen David Adom, National Emergency Medical, Disaster, Ambulance and Blood Bank Service, Ashkelon, Israel; 7 Emergency Department, Azienda Sociosanitaria Ligure 2, Liguria, Italy; 8 ESTES—European Society for Trauma and Emergency Surgery, Disaster and Military Surgery Section, Milan, Italy; 9 Department of Sustainable Development and Ecological Transition, Università del Piemonte Orientale, Vercelli, Italy; 10 Department of Translational Medicine, Università del Piemonte Orientale, Novara, Italy

**Keywords:** Scoping review, Translational science, Mass casualty incident, NIGHTINGALE project, Damage control interventions

## Abstract

**Purpose:**

The European Union Horizon 2020 research and innovation funding program awarded the NIGHTINGALE grant to develop a toolkit to support first responders engaged in prehospital (PH) mass casualty incident (MCI) response. To reach the projects’ objectives, the NIGHTINGALE consortium used a Translational Science (TS) process. The present work is the first TS stage (T1) aimed to extract data relevant for the subsequent modified Delphi study (T2) statements.

**Methods:**

The authors were divided into three work groups (WGs) MCI Triage, PH Life Support and Damage Control (PHLSDC), and PH Processes (PHP). Each WG conducted simultaneous literature searches following the PRISMA extension for scoping reviews. Relevant data were extracted from the included articles and indexed using pre-identified PH MCI response themes and subthemes.

**Results:**

The initial search yielded 925 total references to be considered for title and abstract review (MCI Triage 311, PHLSDC 329, PHP 285), then 483 articles for full reference review (MCI Triage 111, PHLSDC 216, PHP 156), and finally 152 articles for the database extraction process (MCI Triage 27, PHLSDC 37, PHP 88). Most frequent subthemes and novel concepts have been identified as a basis for the elaboration of draft statements for the T2 modified Delphi study.

**Conclusion:**

The three simultaneous scoping reviews allowed the extraction of relevant PH MCI subthemes and novel concepts that will enable the NIGHTINGALE consortium to create scientifically anchored statements in the T2 modified Delphi study.

**Electronic supplementary material:**

The online version of this article (10.1007/s00068-023-02266-0) contains supplementary material, which is available to authorized users.

## Introduction

**Fig. 1:**
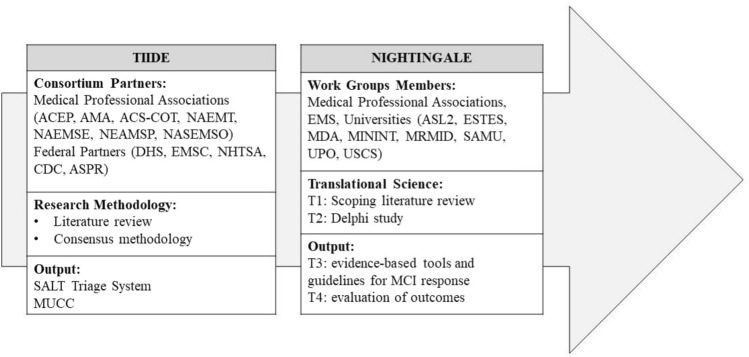
Evolution of the structure and methodology adopted between the TIIDE and NIGHTINGALE project. *ACEP* American College of Emergency Physicians, *ACS-COT* American College of Surgeons-Committee on Trauma, *AMA* American Medical Association, *ASL2* Azienda Sociosanitaria Ligure 2 (Italy), *EMS* Emergency Medical Services, *ESTES* European Society for Trauma and Emergency Surgery, *MCI* Mass Casualty Incidents, *MDA* Magen David Adom—Israel National Emergency Pre-Hospital Medical and Blood Services, *MININT* Ministry of Interior Italy, *MRMID* Swedish International Association for Promotion of Education and Training in Major Incidents and Disasters, *MUCC* Model Uniform Core Criteria for Mass Casualty Triage, *NAEMT* National Association of Emergency Medical Technicians (US), *NAEMSE*  National Association of Emergency Medical System Educators (US), *NAEMSP *National Association of Emergency Medical System Physicians (US), *NASEMSO* National Association of State Emergency Medical System Officials (US), *SAMU* Service d'Aide Médicale Urgente, Paris (France), *SALT* Sort, Assess, Life-Saving interventions, Treatment and/or Transport, *UPO* Università del Piemonte Orientale (Italy), *USCS* Università Cattolica del Sacro Cuore (Italy)

Sudden onset disasters (SODs) and mass casualty incidents (MCIs) from the past have made it evident that collaborative planning activities between public safety, public health, and clinical healthcare providers are essential for successful responses from the whole spectrum of prehospital (PH) response agencies, that must work together to forge and strengthen relationships to produce efficient and effective PH MCI responses [1–3]. As SODs and MCIs continue to increase, there is a real need to develop PH systems that are truly interoperable and integrated. Already in 2007, in response to the need for stronger connections and information exchange between response agencies, the United States Centers for Disease Control and Prevention (CDC) created the Terrorism Injuries: Information, Dissemination, and Exchange (TIIDE) program [4, 5], ultimately aiming to decrease morbidity and mortality from MCIs, including those related to intentional acts of violence and terrorism. One of the projects awarded in the TIIDE was to work toward a national guideline for MCI response, gathering professionals that spanned over the continuum of care: emergency medical services, emergency medical specialists, and trauma surgeons. To achieve this objective, the TIIDE grant consortium partners used consensus methodology based on the best available science to propose the Sort, Assess, Life-Saving interventions, Treatment and/or Transport (SALT) national triage guideline[6] and a model uniform core criteria for mass casualty triage (MUCC) [7]. (Fig. 1).

Despite the literature emerging from the TIIDE project, that spurred further research, discussion, policy, and procedure, current emergency medical services and non-medical civil practitioners involved in PH MCI response often have to rely on complicated or even outdated procedures, multiple protocols or lack of homogeneity in response methods and guidelines, and technology of the past. [8–11]

To this end, the Directorate-General for European Civil Protection and Humanitarian Aid Operations has been emphasizing the need to promote first responders’ preparedness through targeted actions that include the development and update of contingency plans, standard operating procedures, and multi-sector intervention, encouraging involvement of those affected by MCIs and disasters in the design and implementation of such preparedness actions. [12]

In 2020, the European Research Executive Agency (REA), in conjunction with the European Union (EU) Horizon 2020 research and innovation funding program, awarded the Novel InteGrated toolkit for enhanced pre-Hospital life support and Triage IN challenGing And Large Emergencies (NIGHTINGALE) grant to a consortium comprised of a similar wide distribution of PH MCI response agencies and research centers as TIIDE [13] (Fig. 1.). The NIGHTINGALE project features 11 objectives ranging from developing and implementing advanced devices and artificial intelligence, incorporating bystanders into the PH MCI response and addressing ethical challenges, enhancing the collaborative approach across different agencies (Supplementary Table 1). Having acknowledged the evidence produced by the TIIDE project [14], the NIGHTINGALE project seeks to strengthen the existing research in the field of PH MCI and to advance the depth and breadth of PH MCI response guidelines, as advocated by the REA. Therefore, the focus is to upgrade the evaluation of the injured, optimizing life support and damage control procedures and allow a shared response across different MCI responding agencies, including emergency medical services, non-medical civil protection personnel, volunteers, and citizens. To guide the creation of such evidence-based guidelines, the NIGHTINGALE consortium adopted the Translational Science (TS) consensus process that features progressive stages aiming to translate research-informed data into new knowledge in the form of recommendations and guidelines [15] (Fig. 1). As described by Caviglia et al., [16] starting from the TS question (T0), “how to develop, integrate, test, deploy, demonstrate and validate a Novel Integrated Toolkit for Emergency Medical Response which ensures an upgrade to PH MCI response?”, the application of TS in the NIGHTINGALE project entails the identification of current approaches and published data (T1, scoping literature review stage), the use of a consensus methodology as a basis for the development of evidence-based tools and guidelines (T2, modified Delphi), the translation into practice (T3, development of tools and guidelines), and a final evaluation stage (T4, evaluation and outcome assessment) (Fig. 1). In the T1 stage, three simultaneous scoping literature reviews were designed to identify sources and references on MCI Triage, PH Life Support and Damage Control (PHLSDC) interventions, and PH processes (PHP) that could then be interrogated using a defined data extraction tool to determine concepts, theories, and knowledge gaps. [15, 16] Hence, the aim of this work was to map, extract, and synthetize current evidence-based knowledge, gap, and challenges on MCI Triage, PHLSDC, and PHP through three different simultaneous scoping literature reviews, to inform the creation of an initial set of statements for the T2 modified Delphi study.

## Methods

This study describes the structured T1 scoping literature reviews guided by the Preferred Reporting Items for Systematic Reviews and Meta-Analyses extension for scoping reviews (PRISMA-ScR) [17, 18]. To specifically address each component of the PH MCI response, the authors were divided into the three work groups (WGs) of MCI Triage, PHLSDC, and PHP. Each WG included health professionals and researchers with specific expertise in MCI response and patient management. Under the direction of a medical informaticist, the search strategy included only terms relating to or describing sudden onset disaster PH MCI response. The WGs conducted simultaneous searches from November 2021 to January 2022 following the same search term methodology until the search became more specific relating to each WG (MCI Triage, PHLSDC, and PHP), as shown in Table 1.

**Table 1:**
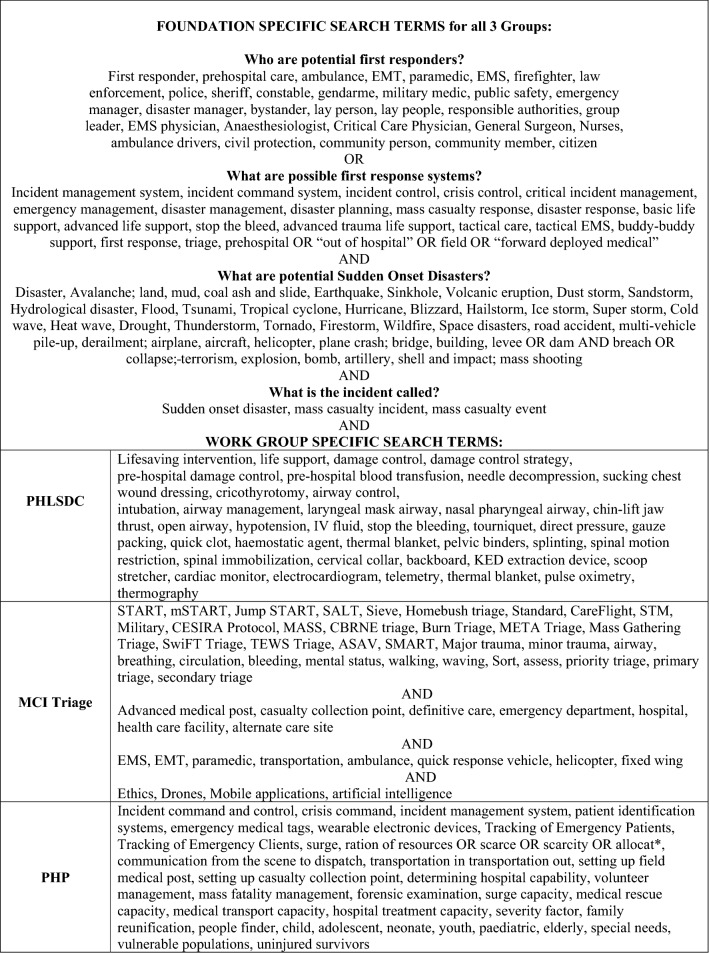
Scoping review search terms

### Eligibility criteria

The search included English-language papers published from January 1983 to October 2021 on PubMed, SCOPUS, CINAHL (Cumulative Index to Nursing and Allied Health Literature, EBSCO, Elton B. Stephens Company, Ipswich, Massachusetts USA), DTIC (Defense Technical Information Center, U.S. Department of Defense), CRI (Emergency Care Research Institute, Plymouth Meeting, Pennsylvania, USA), and PsycInfo (American Psychological Association, Washington D.C., USA). The search terms were adapted for use with other bibliographic databases in combination with database-specific filters for controlled trials, where these were available. An ancestry search was also performed to identify additional references from the bibliography of references retrieved in the searches. The reference manager was EndNote™ X9 and 20 (Clarivate; Philadelphia, Pennsylvania, USA). References that did not meet the inclusion criteria, specifically did not study or report an MCI or MCI exercise, were excluded.

### Search strategy

After each WG conducted its initial search, duplicates were removed. Reviewers in each WG independently screened reference titles and abstracts to determine if inclusion criteria were met. Any disagreement was resolved by discussion within the WG. Each WG removed any reference that did not meet inclusion criteria from further consideration. Subsequently, full texts of included articles were screened, disagreement was resolved by discussion, and references that did not meet inclusion criteria were removed. The remaining included articles for each WG underwent data extraction into an Excel database (Microsoft Corp.; Redmond, Washington, USA) that was developed using themes and subthemes from PH MCI response literature and in compliance with the NIGHTINGALE objectives. This raw data, together with other information gleaned from the full reference review process (such as relevant tables, figures, or writings) constituted the base for the development of the initial set of statements in the T2 modified Delphi stage. Specifically, the data extraction process focused on extracting data that could lead to the creation of relevant statements for the T2 stage by identifying recurrent subjects and innovative concepts that have the potential to improve current practices in PH MCI management but for which validation in clinical settings is still underway.

## Results

The initial search for all 3 WGs yielded 925 references. Following title and abstract review, 483 articles were considered for full text review. Finally, 152 articles were included in the database extraction process: MCI Triage [19–45], PHLSDC [45–81], PHP [30, 43, 82–167]. (Table 2).

**Table 2:** PRISMA statement

	MCI Triage	PHLSDC	PHP
References retrieved via search	304	332	304
Additional via other means	11	2	61
Duplicates discarded	4	5	80
References screened	311	329	285
References discarded	200	113	129
References assessed	111	216	156
References discarded	84	179	68
References data extracted	27	37	88

Different thematic categories and related subthemes were created by each WG (Table 3). The data extraction process drew attention to subthemes frequently mentioned in the included references. Specifically, recurrent subthemes in the MCI Triage WG were the recording of mechanism of injury (*n* = 21), the involvement of bystanders (*n* = 8), the use of basic monitoring in support of PH triage procedures (including cardiac monitoring and pulse oximetry, respectively, mentioned *n* = 12 and *n* = 10 times), re-assessment of MCI casualties though continuous vital signs monitoring (*n* = 14), treatment prioritization (*n* = 15), and evacuation prioritization (*n* = 19) as the main outcomes of the MCI Triage process. Additionally, references included in the MCI Triage data extraction drew attention to the concepts of shock index, pulse pressure, and heart rate variability to be used in the PH assessment of casualties [23, 35].

**Table 3:** Data retrieved from the three groups of MCI Triage, PHLSDC, and PHP, stratified according to identified themes and subthemes

MCI Triage themes, subthemes, (number)	PHLSDC themes, subthemes, (number)	PHP Themes, Subthemes, (Number)
Education Just in time (0) Initial curriculum (3) Maintenance curriculum (1)	Education Just in time (1) Initial curriculum (18) Maintenance curriculum (5)	Education Just in Time (9) Initial Curriculum (3) Maintenance Curriculum (25)
Simulation training Tabletop (1) Live full scale (5) Screen-based (0) Virtual reality (1) Artificial reality (0) Audiovisual (0)	Simulation training Tabletop (5) Live full scale (3) Screen-based (0) Virtual reality (0) Artificial reality (1) Audiovisual (0)	Terminology of MCI processes Mass Casualty Incident (25) Incident management system (3) Disaster response (23) Incident command system (4) Mass casualty response (21) Agreed terminology (4) Critical incident management (1) Incident control (1)
Competency Initial (8) Maintenance (1) Regulatory requirement (2)	Competency Initial (17) Maintenance (10) Regulatory requirement (3)	Competency Initial (3) Maintenance (22) Regulatory requirement (5)
Indications: history (collected over time from scene to definitive care) Mechanism of injury (21) Time since injury (1) By-stander information/intervention/reaction (8) Warm ischemia time (entrapped) (0) Exposure to environment time (warm/cold/wind/water/chemical/smoke) (3) Co-morbid conditions (1) Medications (1) Allergies (0)	Indications: history (collected over time from scene to definitive care) Mechanism of injury (13) Time since injury (3) By-stander information/intervention/reaction (3) Warm ischemia time (entrapped) (1) Exposure to environment time (warm/cold/wind/water/chemical/smoke) (12) Co-morbid conditions (0) Medications (3) Allergies (0)	Policy/Planning framework Government (18) Humanitarian/Non-government (3) Organizational (24) Vulnerable populations (3)
		Activation/incident notification (43) Government request (7) By-stander information/intervention/reaction (3) Organizational activation (23) Surge plans (9) Staff recall (1)
Indications: physical Initial cursory (14) Primary (22) Secondary (13)	Indications: physical Initial cursory (11) Primary (26) Secondary (2)	Command system/authority Government (8) Non-government (2) Organizational (18)
Equipment: standard BP cuff (16) Stethoscope (12) Equipment: resource scarcity (11) IV (start kits, tubing IVF) (5) Meds (2) Bandages (1) Splinting (0) Spinal motion restrictions (0) Other (3)	Equipment to perform PHLSDC Stopping the bleeding (11) Splinting (1) Placing spinal motion restriction (2) Needle decompression (2) Administering antidotes (6) Decontaminating (3) Starting IV's, IVF's (14); blood products (4)	Resource augmentation/ allocation Human resources (15) Equipment (8) Air transport (9) Road transport (4) Mass fatality management (3) Family reunification (1) Field medical post (3) Telemedicine (3) Logistics (10)
Basic monitors used for MCI Triage Cardiac (12) Pulse oximetry (10)	Basic monitors used to perform PHLSDC Cardiac (9) Pulse oximetry (6)	Safety Deployment (5) Decontamination (10) PPE (1) Hazard assessment (15)
Advanced monitors for MCI Triage Ultrasound (1) Physiology monitor (cardiac output, blood vol) (1) Smart watch/bracelet) (2)	Advanced monitors used to perform PHLSDC Ultrasound (2) Physiology monitor (cardiac output, blood vol) (7) Smart watch/bracelet (0)	Casualty Distribution Live time (1) Coordinated/planned (19) Patient tracking (1) Distribution model (19)
Record (patient chart)—electronic record (3) Paper: triage tag, patients' pocket (4) Radio (0) Verbal (0)	Record (patient chart) Paper: triage tag, patients' pocket (5) Radio (4)	Reporting/Documentation Electronic (6) Paper (2) Radio (2)
Reassessment Warm ischemia (0) Compartment syndrome (0) Time on backboard (0) Response to IV’s (3) Response to pain meds (0) Response to antiemetics (0) New complaints (0) Continuous vital signs monitoring (14)	Reassessment Warm ischemia (4) Compartment syndrome (4) Time on backboard (2) Response to IV’s (3) Response to pain meds (0) Response to antiemetics (0) New complaints (2) Continuous vital signs monitoring (8)	Communication/ Situational awareness Social media (5) Radio (10) Telemetry (2) Remote access/ live feed/drone (4)
Outcomes of decisions Treatment prioritization (15) Evacuation prioritization (19)	Outcomes of decisions Treatment of prioritization (13) Airway (8) Bleeding (6) Compartment syndrome (4) Pressure injuries due to prolonged spinal motion restriction (SMR) (0) Emesis with aspiration due to SMR (0) Malignant dysthymia due to hyperkalemia due to crush syndrome (2) Second spinal injury due to lack of SMR or not place SMR (1) Amputation (1) Pul edema due to over fluid resuscitate (1) Other (42)	First Responders All (Volunteer notification, Volunteer activation/management, first responder plans) (15) Volunteer activation/management (3) First Responder plans (3)
Patient tracking Triage tags (3) RFID bracelets (1) Arm bands (1) Smart watch/bracelets (1) Other (2)		
Reporting (within the IMS transfer of care) Electronic (1) Paper: triage tag, patients' pocket (0) Radio (2) Phone (2)	Report (within the IMS transfer of care) Electronic (0) Paper: triage tag, patients' pocket (3) Radio (4) Phone (1)	Recovery/staff care Debrief (2) Staff Welfare (1)

The (N) identifies the number of times a reference discusses the subtheme (some references discuss more than one subtheme). *BP* blood pressure, *IMS* integrated management system, *IV* intravenous, *IVF* intravenous fluid, *MCI* mass casualty incident, *PPE* personal protective equipment, *RFID* radio frequency identification, *SMR* spine motor restriction

Among the 37 references included in the PHLSDC data extraction, the subthemes more frequently identified were the collection of the mechanism of injury (*n* = 13) and exposure to environment time (*n* = 12), the importance of stopping the bleeding maneuvers (*n* = 11) and PH fluid resuscitation (*n* = 14), basic and advanced monitoring to guide PHLSDC interventions, including cardiac monitoring (*n* = 9), pulse oximetry (*n* = 6), and physiological monitoring (*n* = 7), re-assessment of MCI casualties through continuous vital signs monitoring (*n* = 8) and treatment of casualties according to triage prioritization (*n* = 13). Furthermore, the authors identified the following “hot” issues worthy of further attention: PH management of crush syndrome [75], resuscitation of avalanche victims [53, 54], and shared CBRNE treatment protocols [60, 81].

Lastly, the subthemes commonly identified in the PHP data extraction included aspects pertaining to MCI PHP terminology, policy and planning framework, and incident activation/notification. Aspects related to decontamination (*n* = 10) and hazard assessment (*n* = 15) were also frequently mentioned. Furthermore, subthemes related to casualty distribution (either through coordinated/planned methodologies *n* = 19 or an ad hoc distribution model *n* = 19), communication and situational awareness (including the use of social media, radio communication and drones), resource allocation and the integration of technology to support PHP were identified.

## Discussion

The three simultaneous PRISMA scoping reviews were performed in the T1 stage of a TS process, ultimately seeking to advance MCI PH response guidelines in the framework of the EU-funded NIGHTINGALE project. This initial step of mapping current available evidence through the analysis of recurrent subthemes, common practices, and new concepts identified in MCI Triage, PHLSDC, and PHP literature will serve as the basis for the development of three initial sets of statements during the T2 modified Delphi stage. Indeed, results of the three scoping reviews emphasized several aspects that have been recurrently investigated in the MCI literature and for which expert consensus was deemed as needed by the authors.

Despite MCI Triage being the mainstay of initial casualty management, no global consensus or gold standard definition exists across different countries, and most PH practitioners have received training in the initial MCI Triage system favored by their specific agency and jurisdiction [6]. Since numerous attempts have been made toward developing shared guidelines for MCI Triage [5] or in the attempt to create a universal triage tool [168], the NIGHTINGALE project recognizes the necessity to study these efforts as a progression of the previous above mentioned projects. Results of the MCI Triage scoping review determined that recording the mechanism of injury from the initial assessment and as more details emerge over the continuum of care is important for the definitive treatment team [19, 21, 23–26, 29–37, 39, 41–45]. This includes obtaining information not only from EMS staff but also non-medical bystanders who rendered first aid or assisted EMS [28–35], reinforcing the concept that gathering relevant observations from bystanders could support the MCI response efforts, in compliance with the NIGHTINGALE objectives. Information supporting the continuum of care involving the initial and subsequent assessments of vital signs should determine treatment and evacuation prioritization, considering the use of cardiac monitors and pulse oximetry when available. While literature suggests the use of advanced physiologic monitoring and the incorporation of point-of-care ultrasound in the continuum of care [21, 31], it is also evident that initial assessment of MCI casualties should remain quick, practical, easy to remember by all first responders and applicable across different environments including austere settings, thus, to be performed without diagnostic equipment. Appropriate tagging and tracing of MCI casualties remains a pillar of any MCI Triage system adopted, opening the door for innovative solutions and tools possibly fulfilling the triple function of tagging, tracing, and continuous monitoring [27, 42, 44].

References from the PHLSDC scoping review highlighted the need to focus on assessment and treatment guidelines for crush injuries during MCIs [46, 63, 75], as well as for the development of awareness on chemical, biological, radiological, nuclear, and explosive (CRBNE) events, advocating for education, training, and competencies to be developed across all agencies [48, 57, 71, 76, 79, 81]. Similarly, control of major hemorrhages as an integral part of the triage process emerged as a recurrent topic in the included PHLSDC references, with a special emphasis on the role of non-medical bystanders, as specifically stressed by the Hartford Consensus after the Sandy Hook Elementary School mass shooting and by Lesaffre and colleagues after the 2015 Paris attacks [46–48, 50, 51, 61–63, 73, 77, 80, 169]. Overall, attention was given to the importance of continuous real-time monitoring and re-assessment of casualties to guide prioritization of life-saving interventions and transportation, suggesting the possibility to introduce deployable technology, provided it to be quick, reliable, and easy to use [48, 50, 54–56, 63, 69, 75, 76].

Results of the PHP scoping review stressed the importance of a common terminology to be adopted across all agencies working in the same jurisdiction, along with the urgent need to include practices that support gender diversity and are contextual to vulnerable groups and special needs populations [30, 111, 140, 147, 159, 160]. Included references addressed the role of technology in supporting the different PH MCI processes, from inter-agencies communication systems, to telemedicine and information management systems to coordinate the different resources deployed (including human resources, equipment, and vehicles) and to distribute MCI casualties in different health facilities [90, 92, 99, 146, 160]. Data supported that standard trauma transportation decisions and overall MCI coordination may have to be adapted in context to the hazard impact and health system capacity, taking into account the possibility of CBRNE threats demanding from patient decontamination and personnel self-care [86, 88, 89, 100, 102, 103, 108, 136]. Additionally, from the results emerged that frequently definitive care may require alternative care sites to include field hospitals, adapted structures such as conventions centers, schools, or religious buildings like churches and mosques that have the capacity and capability to attend the injured [105, 131, 151]. The concept of enhancing situational awareness through available technology (such as drones) to better guide critical decision-making during MCIs emerged in some of the included references, especially in remote areas with access constraints [90, 92, 99, 109, 117, 129, 133, 139, 146, 160]. Moreover, the role of spontaneous volunteers and bystanders both in assisting relief efforts [88, 107, 133, 134, 141, 144, 166] and in providing information through social media that could be used for rapid situation assessment [129] consistently emerges in the included references. Lastly, a recurrent notion highlighted in the included references was the need for a structured MCI plan able to regulate the use of technical advances, to foster education and training competences across different agencies, to allow for structured debriefing in a collaborative manner, and to promote the use of key performance indicators to evaluate and improve the response [83–86, 91, 98, 100–104, 107, 109–112, 116, 119, 120, 122, 124, 131, 135, 137, 142, 145, 148, 153, 158, 160, 164, 166].

### Limitations

A scoping review intends to capture all included peer-reviewed publications as well as the ancestry publications that were cited in these publications. The Grey literature presents challenges to obtain relevant references and all attempts were made to include these references, but some may not have been obtained. There is no way to know if these potential missing references would have made a difference in the available information to extract data. The raw data extracted by WG members and the work group lead are intended to undergo further analysis within each work group to create the initial T2 modified Delphi statements. This data extraction process, though intended to be encompassing and comprehensive, may not have captured relevant data due to the thematic approach of the database. There is no way to know if relevant data were not extracted but the secondary data retained by each work group member as they read each full reference article provide additional information for the statement creation process.

## Conclusion

The progression of the science to critically examine the PH MCI response peer-reviewed medical literature and other sources has enabled the NIGHTINGALE partners to methodically obtain raw data and secure relevant tables, figures, or writings that will contribute to each WG’s creation of the initial T2 modified Delphi study statements. Once submitted to experts, statements that achieve consensus will be used to define guidelines and recommendation, in compliance with the objectives of the NIGHTINGALE project.

## Electronic supplementary material

Below is the link to the electronic supplementary material.

Supplementary file1 (DOCX 15 kb)

## Data Availability

The authors confirm that the data supporting the findings of this study are available within the article and its supplementary materials.
