# Supplementary material for: Advancing the scientific study of prehospital mass casualty response through a Translational Science process: the T1 scoping literature review stage
Source: Eur J Trauma Emerg Surg. 2023 Apr 15;49(4):1647–60. doi: 10.1007/s00068-023-02266-0 (PMC10449715; doi:10.1007/s00068-023-02266-0)
Supplement: Supplementary file 1 — Supplementary file1 (DOCX 15 kb) [file 68_2023_2266_MOESM1_ESM.docx]

**Supplementary material**

| **NIGHTINGALE OBJECTIVES** |
| --- |
| **OBJ-1: To increase EU resilience against natural and man-made attacks by augmenting medical response capabilities in all types of disasters (multi-hazard approach).** |
| **OBJ-2: To deliver a Novel Integrated Toolkit for Emergency Medical Response (NIT-MR) extensively validated by a large number of emergency medical services, non-medical civil protection agencies, citizens, volunteers and technology providers in the field facilitating systematic training, standardisation and certification.** |
| **OBJ-3: To deliver a step change to pre-hospital life support and triage, not only by enhancing medical operational capacities for digital identification and traceability of patients and actions, fast diagnosis and prognosis and continuous monitoring of vital signs and enablement of accurate classification, but notably by optimising current procedures, methods and guidelines of emergency medical and other involved actors through defining common denominators among responders, identifying gaps and challenges in medical response and materialising lessons learned on damage control and mass casualty handling from the military domain and from past events.** |
| **OBJ-4: To upgrade Triage and pre-hospital life support by developing a suite of interconnected wearable technologies, sensors and mobile applications which enhance, via Artificial Intelligence, fast diagnosis and prognosis, classification and damage control processes of emergency medical personnel, empower efficient tasking of non-medical civil protection actors and volunteers and allow digital identification, traceability and monitoring of patients and response actions during mass casualty incident handling.** |
| **OBJ-5: To optimise transportation and medical and civil protection resources availabilities and utilisation (e.g. personnel, vehicles, hospital beds and capacities, medical equipment, supporting equipment, etc.) by developing a set of ancillary devices and platforms, empowered by Artificial Intelligence based decision support functions, rapidly and autonomously deployed in the field which gather information, enhance awareness, localise personnel and their assets, and optimise communication between teams and patients.** |
| **OBJ-6: To fuse all available information under an integrated framework, and develop an advanced C3 (Command, Control & Coordination) and Incident Management for MCI (IMS), providing the Common Operating Picture to all types of response units, advancing coordination and cross-team collaboration.** |
| **OBJ-7: To convey the COP to the Responders, by exploiting recent advances in Augmented Reality, and developing a set of mobile Apps and that allow the FR units to better coordinate tactical and operational response.** |
| **OBJ-8: To increase public safety, civil protection and mass casualty incidents handling by greatly improving the capabilities of EU medical response and non-medical civil protection units.** |
| **OBJ-9: To allow cross-domain and cross-country medical and civil protection response team collaboration overcoming administrative, disciplinary and political barriers through technology and commonly agreed operational practices.** |
| **OBJ-10: To ensure legal, societal, ethical, security considerations and relevant impact assessments, advancing NIGHTINGALE sustainability, acceptance, credibility and adoptability, are fully embedded by design to its implementation.** |
| **OBJ-11: To engage, inclusively, all relevant emergency medical services (incl. EU and Internationally wide emergency medicine organisations) and non-medical civil protection agencies (fire brigades, police and search and rescue personnel, but also volunteers and citizens) as well as technology stakeholders in the Action maximising market penetration and exploitation of the Toolkit and the defined methods and guidelines, hence facilitating adoption by the FR community.** |

**Suppl Table 1. NIGHTINGALE (6) Objectives**
